# Association of arthritis and total joint arthroplasty with self‐reported function in former professional American‐style football players

**DOI:** 10.1002/pmrj.70058

**Published:** 2025-12-27

**Authors:** Michelle M. Bruneau, Rachel Grashow, Michael Leung, Alicia J. Whittington, Herman A. Taylor, Marc G. Weisskopf, Frank E. Speizer, Ross Zafonte, Adam S. Tenforde

**Affiliations:** ^1^ Department of Physical Medicine and Rehabilitation Harvard Medical School, Spaulding Rehabilitation Hospital Charlestown Massachusetts USA; ^2^ Football Players Health Study at Harvard University, Harvard Medical School Boston Massachusetts USA; ^3^ Department of Environmental Health Harvard T.H. Chan School of Public Health Boston Massachusetts USA; ^4^ Morehouse School of Medicine Atlanta Georgia USA; ^5^ Channing Division of Network Medicine, Brigham and Women's Hospital Boston Massachusetts USA; ^6^ University of Missouri School of Medicine Columbia Missouri USA

## Abstract

**Background:**

Participation in American‐style football (ASF) results in trauma‐related concerns including joint injuries. Limited work has described arthritis and knee and hip total joint arthroplasty (TJA) in this population. The association of these conditions to pain interference and physical and mental function has not been well described.

**Objectives:**

1. To characterize demographic, football, and health‐related factors in former ASF players associated with arthritis and knee or hip TJA. 2. To investigate the association of arthritis and knee or hip TJA with pain interference and physical and mental function.

**Study Design:**

Cross‐sectional cohort study.

**Setting:**

Academic medical multisite hospital system.

**Participants:**

Former ASF players who played professionally from 1960 to 2019.

**Assessment of Risk Factors:**

Self‐completed standardized questionnaires.

**Main Outcome Measures:**

Surveys included self‐reported arthritis and knee and hip TJA, Patient‐Reported Outcome Measure Instrument Scale (PROMIS), physical function and mental function, and pain interference scales. Multivariable logistic regression models assessed the association between demographic, football‐related, and health characteristics with arthritis and knee and hip TJA. Multivariable linear regression models evaluated the association between arthritis and knee and hip TJA with pain interference and physical and mental function.

**Results:**

In 4189 former ASF players (average and SD: 51.8 ± 14.4 years old) over half (*n* = 2237, 53.4%) had arthritis (*n* = 1547) or knee or hip TJA (*n* = 690). Both arthritis and knee and hip TJA were more common in those who were older, with higher body mass index, and prior surgery during playing years and were linemen (*p* < .05). Additionally, arthritis was more common in players who were never married (*p* = .01) and with higher concussion symptoms score (*p* < .001). Knee and hip TJA were more common in players who self‐identified as White (*p* < .001). Both arthritis and knee and hip TJA were associated with greater pain interference and reduced physical function (all *p* < .001) but not with mental function.

**Conclusion:**

More than half of former ASF players reported arthritis or knee or hip TJA. Higher pain interference and reduced physical function in former ASF players with arthritis and knee or hip TJA highlight the importance of advancing strategies to prevent and treat joint conditions.

## INTRODUCTION

American‐style football (ASF) is a popular collision sport with recognized health concerns related to participation. Although both neurocognitive conditions and cardiovascular disease have been characterized in former ASF players,^1^, ^2^, ^3^, ^4^, ^5^ limited work has described musculoskeletal impairments including joint disease and surgery.^6^ Arthritis is the most common form of joint disease in the general populations^7^ and former athletes,^8^, ^9^ including former ASF players.^6^ Although prior work in this population has described arthritis as related to older ages and previous lower extremity injury,^4^, ^6^ modifiable risk factors to reduce arthritis in current and former ASF players have yet to be identified.^6^, ^10^, ^11^, ^12^

Symptomatic arthritis is a painful and disabling condition that may ultimately result in total joint arthroplasty (TJA) to restore function and improve quality of life. Former ASF players are more likely to report knee or hip TJA compared to the U.S. general population, when stratified by age.^13^, ^14^ Prior studies in former ASF players identified older age, football‐related characteristics, and health factors as risk factors for knee and hip TJA.^10^ No prior study has characterized both arthritis and knee or hip TJA among former ASF players and how these factors may be interrelated. Such information could identify former ASF players at risk for arthritis and knee or hip TJA and guide future longitudinal studies to investigate risk factors for arthritis and knee and hip TJA in this population.

Arthritis contributes to higher pain severity and reduced physical function in the general population and former athletes.^8^, ^15^ Prior work has explored mental health, physical function, and pain interference in former ASF players.^16^, ^17^, ^18^ Pain interference is defined as a measure of the extent to which pain hinders engagement with physical, cognitive, emotional, and recreational activities, as well as sleep and enjoyment in life.^19^ Across medical conditions, chronic pain was associated with the greatest limitation in physical and mental function in former ASF players. However, the contribution of arthritis and knee and hip TJA to chronic pain has not been adequately characterized in former ASF players. Although many report improved health outcomes, knee and hip TJA may not restore functional status and a prior study reported greater pain severity and reduced function in 30% of patients at 6 months following knee TJA.^20^

The purpose of this study was to (1) identify independent associations between demographic, football‐related, and health characteristics with arthritis and hip or knee TJA in former ASF players, and (2) explore the association of arthritis and hip or knee TJA with pain interference and physical and mental function. We hypothesized that (1) arthritis and hip or knee TJA would be associated with older age, higher body mass index (BMI), playing position, longer career duration, and previous surgeries; and (2) arthritis and hip or knee TJA would be associated with higher pain interference and reduced physical and mental function in former ASF players. Understanding former ASF players affected with arthritis and hip or knee TJA may provide valuable insight into factors associated with these conditions and promote future longitudinal studies to investigate risk factors for arthritis and hip or knee TJA in this population.

## METHODS

### Participants

The Football Players Health Study at Harvard University is an ongoing investigation designed to understand the health of former ASF players.^21^ Eligible participants were former ASF players who played in the National Football League since 1960. Participants could complete surveys using electronic surveys on Research Electronic Data Capture (REDCap) or via mail contact information provided by the National Football League Players Association to complete the survey using Scantron (Tustin, CA). The study was approved by the Harvard T.H. Chan School of Public Health and participants provided written informed consent in accordance with institutional policies. Additional recruitment details are available in previous publications.^21^

### Demographics

Participants were invited to complete a self‐administered questionnaire that included questions on demographics (eg, age, race, and domestic status), football exposures, health status, and current function.

### Football exposure

Football exposure variables included the following: (1) “How many seasons did you actively play professional football?” and (2) “Select the positions that you most often played during your professional football career from the following: offensive line, defensive line, linebacker, defensive back, running back, wide receiver, tight end, quarterback, kicker/punter, special teams.” Given prior work describing that linemen have higher prevalence of hip TJA,^10^ player position was evaluated and categorized as lineman (defensive line, linebacker and offensive line) or nonlineman (defensive back, kicker/punter, quarterback, running back, tight end, wide receiver and special teams) for study analyses. Finally, concussion symptoms score (CSS) was used to assess participants' history of repetitive head injury. The CSS is calculated based on questions related to the frequency of 10 football‐related head injury symptoms accrued during youth, collegiate, and professional football play that were summed across items.^22^, ^23^ The CSS has been shown to be consistent across multiple years and is unrelated to years since play, age of the participant, and current health status.^24^ CSS was included as a variable of interest based on previous literature that has identified an association between concussion and other musculoskeletal injuries^25^, ^26^, ^27^ and thus we were interested in investigating this association with arthritis and knee or hip TJA in former professional ASF players.

### Health‐related factors

We calculated current BMI from each participant's reported current height and weight as higher BMI has been associated with TJA in other work.^28^, ^29^ Demographic variables were also assessed including marital status and race as these factors may influence health outcomes. The number of surgeries (neck, back, knee, anterior cruciate ligament [ACL] reconstruction, ankle, shoulder, or hand) during their playing years was reported. The presence of arthritis and/or hip or knee TJA was identified from responses to the following questions: “Has a health care provider ever told you that you have arthritis?” and “Since leaving active professional football, have you had any of the following surgical procedures: (knee joint replacement) or (hip joint replacement)?”

To understand the association of arthritis and TJA in this population compared to those without either condition, physical and mental function and pain interference outcome measures at time of survey completion were assessed with standardized questions that included the Patient‐Reported Outcome Measure Instrument Scale (PROMIS) Physical Function v2.0 – Short Form (6b) and the PROMIS Global Health Scale v1.2. The PROMIS Physical Function^30^ captures the perceived difficulty of daily physical tasks such as vacuuming, yard work, and house cleaning. The response scale ranges from 0 (“without any difficulty”) to 4 (“unable to do”). The PROMIS Global Health Scale v1.2 is divided into two subsets: Global Mental Health and Global Physical Health.^30^ The Global Mental Health score contains four questions that evaluate mental function, emotional problems, life quality, and social activities and relationships, with responses varying from 0 (“poor”) to 4 (“excellent”). The Global Physical Health subset addresses physical health, function, pain, and fatigue, using the same response scale.^30^, ^31^ Pain interference was assessed using the PROMIS Pain Interference Scale 6b, which includes six questions related to pain interference with daily living, recreational activities, and enjoyment in life.^19^, ^30^ Responses ranged from 1 (“not at all”) to 5 (“very much”).^19^

### Statistical analyses

Descriptive statistics were reported as mean ± SD. Chi‐square tests were used to assess differences in demographics, football characteristics, and health in those with and without arthritis and/or TJA. When categorical data were missing, we assigned the category to “missing.” We used a “complete case” analysis approach whereby observations with missing continuous data were excluded because we determined that the percentage of participants with any missing data was 3.7%. We assume that these were missing at random (ie, missingness is random conditional on the covariates), but in the event that they are missing not at random (ie, there are systematic differences between those with and without missing data), their impact on our associations would be minimal. We used logistic regression models to estimate odds ratios (OR) and 95% confidence intervals (CI) for arthritis and knee or hip TJA associated with demographic, health, and football‐related characteristics. The models included the following variables based on a priori factors associated with other populations:^10^, ^32^, ^33^ age, race, current BMI, domestic status, playing position (linemen vs. nonlinemen), career duration, active surgery during playing years, and concussion symptoms score. Career duration was assessed as categorical as prior work has often seen nonlinear associations with this variable.^1^ Because the most common reason to have knee or hip TJA is arthritis,^34^, ^35^, ^36^ we imputed “yes” for having arthritis for all participants who had reported TJA, creating a single categorical variable of “TJA.”

Recognizing the development of most orthopedic and health conditions is multifactorial, we used multivariable linear regression models assessed associations in former ASF players with arthritis or knee or hip TJA to assess pain interference, physical and mental function. The model controlled for factors related to demographics, function and quality of life including age, race, domestic status, employment status, sleep apnea, current BMI, years since play, career duration, playing position, concussion symptoms score, surgery during playing years, surgery after leaving professional football, and current pain medication prescription. For all models, any missing values were treated as missing at random without imputation using a missing indicator. Any missing values were excluded from the model. Statistics were calculated using R Language for Statistical Computing.^37^ Significance was defined as *p* < .05.

## RESULTS

### Participant characteristics

Among the 4189 (33.9% enrolled) who completed the initial questionnaire between January 30, 2015 and November 29, 2022 reported on previously^1^, the mean current age of former ASF players was 52 ± 12.8 years and nearly 53% had a current BMI >30 kg/m^2^ (Table 1). Most participants (*n* = 2237, 53.4%) reported arthritis (*n* = 1547) or knee or hip TJA (*n* = 690). Those with arthritis and knee or hip TJA were older and a greater proportion had a BMI >30 kg/m^2^ at time of survey completion. Although the racial demographic of those reporting arthritis was similar to the full cohort, a larger portion of former ASF players who reported knee or hip TJA were White. Over half of former players with arthritis were linemen (52.5%). Surgery during playing years was more common in those with arthritis or knee or hip TJA. Additionally, those with arthritis had a higher concussion symptoms score. Those with arthritis (43.60 ± 8.21) and knee or hip TJA (43.00 ± 8.05) reported similar physical function, both reporting worse function on the PROMIS Global Physical Health measures than those without arthritis or knee or hip TJA (49.90 ± 8.44). This trend was similar for both mental function (PROMIS Global Mental Health) and pain interference (PROMIS Pain Interference) (Table 1).

**TABLE 1:** Descriptive characteristics of former American‐style football players.

Variable	No arthritis or total joint arthroplasty (*n* = 1952)	Arthritis only (*n* = 1547)	Knee or hip total joint arthroplasty (*n* = 690)	Total (*n* = 4189)
Age, y, mean ± SD	46.5 ± 13.7	52.6 ± 12.8	64.9 ± 10.3	51.8 ± 14.4
BMI, *N* (%)				
<25	117 (6.0%)	74 (4.8%)	32 (4.6%)256	223 (5.3%)
25–30	883 (45.2%)	583 (37.7%)	(37.1%)	1722 (41.1%)
>30	936 (48.0%)	879 (56.8%)	399 (57.8%)	2214 (52.9%)
Missing	16 (0.8%)	11 (0.7%)	3 (0.4%)	30 (0.7%)
Race, *N* (%)				
Black	848 (43.4%)	626 (40.5%)	160 (23.2%)	1634 (39.0%)
White	1016 (52.0%)	860 (55.6%)	500 (72.5%)	2376 (56.7%)
Other	66 (3.4%)	44 (2.8%)	16 (2.3%)	126 (3.0%
Missing	22 (1.1%)	17 (1.1%)	14 (2.0%)	53 (1.3%)
Domestic status, *N* (%)				
Living with partner	99 (5.1%)	69 (4.5%)	25 (3.7%)	193 (4.6%)
Married	1407 (72.7%)	1193 (77.8%)	560 (82.0%)	3160 (76.1%)
Never married	212 (11.0%)	83 (5.4%)	19 (2.8%)	314 (7.6%)
Separated/divorced	208 (10.7%)	171 (11.2%)	65 (9.5%)	444 (10.7%)
Widowed	10 (0.5%)	17 (1.1%)	14 (2.0%)	41 (1.0%)
Missing	16	14	7	37
Career duration mean ± SD	5.62 ± 3.50	7.1 ± 3.9	7.2 ± 3.5	6.7 ± 3.9
Lineman status, N (%)				
Lineman	283 (41.0%)	52 (52.5%)	559 (36.1%)	1420 (33.9%)
Not lineman	407 (59.0%)	47 (47.5%)	988 (63.9%)	2769 (66.1%)
Concussion symptoms score mean ± SD	27.01 ± 25.62	36.87 ± 28.90	27.63 ± 24.94	30.74 ± 27.17
Surgery during playing years	1.11 ± 1.14	1.58 ± 1.29	1.58 ± 1.25	1.36 ± 1.24
Mean ± SD				
PROMIS Global Physical Health^a^	49.90 ± 8.44	43.60 ± 8.21	43.00 ± 8.05	46.42 ± 8.90
PROMIS Global Mental Health^a^	47.90 ± 9.94	44.00 ± 10.20	46.40 ± 9.95	46.22 ± 10.21
PROMIS Pain Interference^b^	52.90 ± 8.58	59.00 ± 8.00	58.00 ± 8.01	55.98 ± 8.78

Abbreviations: BMI, body mass index; *N*%, Frequency percentage; PROMIS, Patient‐Reported Outcome Measure Instrument Scale.

^a^ PROMIS Global Physical and Mental Health: higher scores associated with better function.

^b^ PROMIS Pain Interference: higher scores associated with higher pain interference.

### Factors associated with arthritis

Out of the 4139 former players enrolled, 120 had missing outcome values (arthritis and knee or hip TJA) and could not be included, resulting in 4019 participants. Among the remaining participants, 3.7% of the players were missing at least one covariate or exposure metric. Given that this value was <5%, we used a complete case analysis approach, and dropped those individuals with missing data from the analytic dataset.^38^ Higher current BMI (OR = 1.04, [95% CI: 1.03–1.06]) and older age (OR per SD = 1.95 [95% CI: 1.81–2.11]) were associated with higher likelihood of arthritis. Former ASF players who were never married (OR = 0.70 [95% CI: 0.53–0.93]) were less likely to report arthritis. Football‐related factors related to arthritis include being a lineman (OR = 1.20 [95% CI: 1.02–1.41]), surgery during playing years (OR = 1.39 [95% CI: 1.31–1.47]), and higher concussion symptoms score (OR per SD = 1.43 [95% CI: 1.33–1.54]); (Table 2).

**TABLE 2:** Odds ratios for arthritis and arthroplasty by demographic, football‐related, and health factors.

	Arthritis		Knee or hip total joint arthroplasty	
	*OR (95% CI)*	*p value*	*OR (95% CI)*	*p value*
Age	1.95 (1.81–2.11)	**<.001** ***	4.29 (3.76–4.92)	**<.001** ***
Race (Black)	0.92 (0.79–1.07)	.27	0.67 (0.53–0.84)	**<.001** **
Current BMI	1.04 (1.03–1.06)	**<.001** ***	1.05 (1.03–1.07)	**<.001** ***
Domestic status				
Living with partner	1.02 (0.73,1.41)	.93	1.15 (0.68,1.86)	.59
Never married	0.70 (0.53–0.93)	.**01** *	1.21 (0.70–2.00)	.47
Separated/divorced	0.81 (0.65–1.01)	.07	0.85 (0.62–1.16)	.32
Widowed	1.09 (0.54–2.30)	.81	0.87 (0.40–1.82)	.72
Concussion symptoms score	1.43 (1.33–1.54)	**<.001** ***	0.97 (0.87–1.07)	.57
Lineman	1.20 (1.02–1.42)	.**02** *	1.28 (1.05–1.59)	.**02** *
Surgery during playing years	1.39 (1.31–1.47)	**<.001** ***	1.30 (1.20–1.40)	**<.001** ***
Career duration				
3–6 seasons	1.08 (0.78–1.34)	.47	1.15 (0.82–1.62)	.42
>6 seasons	1.23 (0.99–1.52)	.66	1.15 (0.83–1.61)	.42

*Note*: Displays odds ratio for arthritis and knee or hip total joint arthroplasty determined based on logistic regression models that included the following variables: age, race, current BMI, domestic status, playing position (lineman vs nonlineman), career duration, active surgery during playing years, and concussion symptoms score. Bolded values indicate statistical significance.

Abbreviations: BMI, body mass index; CI, confidence intervals; OR, odds ratio.

* *Significance p < .05*.

** *Significance p < .01*.

*** *Significance p < .001*.

### Factors associated with knee or hip total joint arthroplasty

Similar to arthritis, higher BMI (OR = 1.05 [95% CI: 1.03–1.07]) and older age (OR per SD = 4.29 [95% CI: 3.76, 4.92]) were associated with TJA. Linemen (OR = 1.28 [95% CI: 1.05–1.59]) and those who had surgery during playing years (OR = 1.30 [95% CI: 1.20–1.40]) were more likely to have TJA. In contrast to arthritis, former ASF players who identified as Black were less likely to report TJA than White former players (OR = 0.67 [95% CI: 0.53–0.84]). A higher concussion symptoms score was not associated with TJA (*p* > .05; Table 2).

### Association between arthritis and knee or hip TJA with pain interference and physical and mental function

**FIGURE 1:**
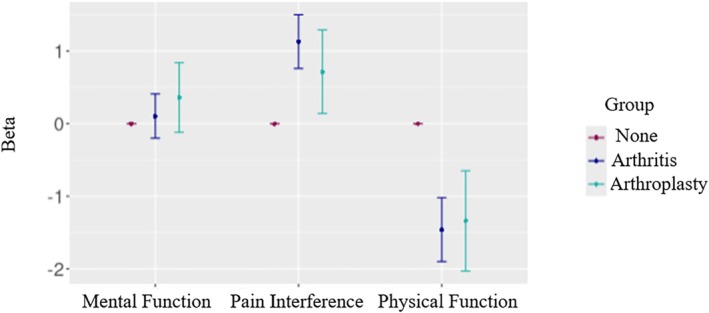
Associations of arthritis and arthroplasty with mental and physical function and pain interference. Difference in mental function, pain interference, and physical function scores among those with arthritis or arthroplasty compared to those without. Vertical lines represent 95% confidence intervals. The models controlled for age, race, domestic status, employment status, sleep apnea, current BMI, years since play, career duration, playing position, concussion symptoms score, surgery during playing years, surgery after leaving professional football, current pain medication prescription, and the PROMIS Global Health Scale. Confidence intervals in the positive range for pain interference means it scores high in pain, representing higher pain levels, while confidence in the negative range for physical function means reduced physical function. BMI, body mass index; PROMIS, Patient‐Reported Outcome Measure Instrument Scale.

Higher pain interference was associated with both arthritis (β = 1.13 [95% CI: 0.76–1.50], *p* < .001) and TJA (β = 0.71 [95% CI: 0.14–1.29], *p* = .02; Figure 1). Reduced physical function was associated with arthritis (β = −1.46 [95% CI: −1.90 to −1.02], *p* < .001) and TJA (β = −1.34 [95% CI: −2.03 to −0.65], *p* < .001). In contrast, there was no association of mental function and arthritis (β = 0.10 [95% CI: −0.20 to 0.41], *p* = .51) or knee or hip TJA (β = 0.36 [95% CI: −0.12 to 0.84], *p* = .14).

## DISCUSSION

The purpose of this study was to characterize former ASF players with arthritis and knee or hip TJA. We explored historical factors related to participation in ASF and health factors reported at survey completion to investigate factors more strongly associated with developing arthritis or prior knee or hip TJA and how those former ASF players with arthritis/knee or hip TJA were functioning measured using pain interference and physical and mental function. Higher pain interference and reduced physical function were observed in those with arthritis and TJA whereas there was no association observed with differences in mental function. The association of higher pain interference in former ASF players with arthritis is consistent with prior work in former NFL players and impairments observed in other athlete populations.^18^, ^39^, ^40^ For example, pain interference, measured using the Short Form Health Survey and the Patient‐Reported Outcomes Measurement Information System to assess how pain affects various aspects of a player's life, increased in 338 former ASF players over 19 years after retiring from football, and arthritis was strongly associated with overall pain interference levels and trajectory.^18^ The current study demonstrates an association of pain interference with arthritis in a larger cohort. However, the explanation for higher pain interference in former ASF players with knee or hip TJA cannot be determined by cross‐sectional study design. One plausible explanation is that those with TJA have a greater burden of arthritis beyond the joint with arthroplasty and that other factors such as chronic pain should be considered. Likewise, impaired physical function was observed with arthritis in our cohort, findings that suggest former athletes may experience findings similar to the general population^41^ and other athletes.^42^, ^43^, ^44^ For example, in the general population, arthritis is associated with limitations in physical function likely due to pain, which can negatively affect mental function and quality of life.^45^ In contrast to prior work characterizing chronic pain in former ASF players,^3^ arthritis and knee or hip TJA were not associated with impaired mental function. The reason for this is unknown; however, the cohort with chronic pain was defined as having been prescribed medication to treat pain, as defined in previous studies.^46^, ^47^ Our findings bring attention to the importance of understanding arthritis and knee or hip TJA along with exploring further pain management strategies in this population.

We identified health characteristics and demographic factors associated with arthritis and knee or hip TJA. Similar to the general population,^48^, ^49^ older age was associated with both arthritis and knee or hip TJA in former ASF players. Higher BMI was associated with both arthritis and knee or hip TJA, findings that have been reported in other athletes,^29^ including Olympic athletes with arthritis.^28^ Former ASF players who were never married were less likely to report arthritis. Additionally, we observed race differences in the proportion of former players with knee or hip TJA. Although a similar proportion of former ASF players reported arthritis, knee or hip TJA was disproportionately higher in those who identified as White. Although the source of this disparity among former ASF players is not well understood, studies conducted in the general population have found that Black patients have lower TJA utilization rate and worse outcomes post TJA, including more emergency room visits and higher readmission rates, than White patients.^33^, ^50^ Health care access, education, affordability, communication, navigating the health system, and provider bias have been identified as potential drivers of these disparities.^51^ Studies suggest that social determinants of health, the nonmedical factors, may drive 50% of modifiable risk factors that determine how a patient fares a surgery.^50^ Previous findings by the Football Players Health Study have demonstrated that former professional ASF players experience disparities by race in broad areas of health, even after controlling for football exposures, such as playing position, career length, BMI, and pain medication use.^52^ Although speculative, it is possible that former ASF players who are unmarried may not seek medical care and receive proper diagnosis. Collectively, this suggests that a career in ASF is not protective from health disparities observed in the general population.

Several characteristics related to football exposure were associated with both arthritis and knee or hip TJA. Playing lineman and surgery during playing years were correlated with a greater burden of both arthritis and knee or hip TJA. Style of play, such as quick bursts of movement required to accelerate from a three‐point stance squatting position required of linemen,^53^ could place unique demands on the knees and hips. The sport involves repetitive collisions that are transmitted to the full body including joints and the head. Notably, higher prior concussion burden was associated with arthritis. Although history of concussion has been associated with ACL injuries in other athletic populations,^25^, ^26^, ^27^ we are unaware of any current studies that have assessed the relationship between arthritis and knee or hip TJA with exposure to repetitive head injury in former professional ASF players. Prior studies have identified an association between concussion and altered proprioception,^54^, ^55^, ^56^ and thus it is plausible that altered proprioception may contribute to increased risk of ACL or lower extremity injury that affects the joints and leads to subsequent arthritis in former ASF players. Additionally, the concussion symptom score could simply be correlated with orthopedic injuries acquired during playing years that might affect later risk of arthritis or knee or hip TJA independent of a specific relation with head trauma. Our findings combined with those of previous studies^10^ suggest a relationship between football exposure and arthritis with knee or hip TJA in former professional ASF players. Arthritis may be more common in former ASF players from early arthritis related to posttraumatic osteoarthritis and expands on work in a prior study on chronic disease burden in this population.^4^ Furthermore, the prevalence of knee or hip TJA may be related to those who are willing to have surgery rather than qualifying as a TJA candidate alone.

We acknowledge several limitations in our study. Players self‐reported their health including answering the question, “Have you been told by a health care provider that you have arthritis?”, and the question does not specify the type, severity, or location of arthritis. Additionally, only hip and knee TJA were included, as we did not inquire about other types of joint replacements in the questionnaire. Therefore, findings cannot be generalized to joint replacements in other body regions and future studies in this population should ask about all joint replacements. The cross‐sectional study design limits understanding the mechanisms and time course for arthritis and temporal relationship of response to both arthritis and knee or hip TJA to determine causality. The inability to ascertain additional variables (ie, reasons for not having surgery or recommendations by health care providers regarding surgery) limits insight into those with arthritis who elect to have a knee or hip TJA. Although this study represents the largest cohort of living former ASF players, we cannot verify the population is representative of the full population of former professional football players; however, many of the characteristics of our sample match those of the entire population.^21^ We assume any missing data were missing at random. Even if there were systematic differences between those who did and did not answer the covariate, football exposure, or TJA and arthritis questions (ie, missing not at random), they would not substantially affect our associations given the overall low rate of missingness in our data (<3.7%). Finally, we do not have information on former players' health insurance access, which would allow us to contextualize access to care and provide further insight on potential social factors associated with arthritis.

In conclusion, we identified demographic, football exposure, and health factors associated with arthritis and knee or hip TJA in former professional ASF players. Future prospective studies should investigate these variables as potentially modifiable risk factors for arthritis and knee or hip TJA in this population. Our study adds to limited work^10^ and represents the largest longitudinal cohort of living former NFL players that assesses health. We identified higher pain interference and worse physical function associated with arthritis and knee or hip TJA. However, there was no clear association with mental function. Our findings have important clinical implications for providers who are caring for former ASF players with arthritis and knee or hip TJA. Future studies should explore the systemic factors and social determinants of health that may influence the need for knee or hip TJA in ASF players. Understanding why former ASF player who were never married were less likely to have a diagnosis of arthritis and the observation that former Black players were less likely to undergo knee or hip TJA highlights potential barriers to receiving optimal management of arthritis in this population. Collectively, this suggests the value of further work in this population to guide appropriate care of former ASF players.

## DISCLOSURE

None of the authors report a conflict of interest regarding the manuscript being submitted to *PM&R*.

## Data Availability

The data that support the findings of this study are available from the corresponding author upon reasonable request.
